# Randomized Trial of Sequential Compression Versus Ankle-Calf Movement to Increase Femoral Venous Velocity

**DOI:** 10.7759/cureus.48070

**Published:** 2023-10-31

**Authors:** Saurabh Kumar, Negin Azadi, Donald Emerson, Joseph Santoso

**Affiliations:** 1 Obstetrics and Gynecology, University of California, Riverside, USA; 2 Obstetrics and Gynecology, Meharry Medical College, Nashville, USA; 3 Radiology, Methodist Le Bonheur Healthcare, Memphis, USA; 4 Obstetrics and Gynecology, Gynecologic Oncology, Baptist Medical Group, Memphis, USA

**Keywords:** postoperative care, sequential compression device, compression stockings, ankle calf flexion movement, venous thromboembolism (vte)

## Abstract

Objective

In postoperative patients with a high risk of bleeding, sequential compression devices (SCD) and ambulation are effective methods to reduce venous thromboembolic (VTE) risks. High leg venous flow decreases VTE risk. We postulated that ankle flexion and extension (AFE) while in bed increased leg venous flow velocity as well. We wished to compare the effectiveness of SCD versus AFE in increasing leg venous velocity.

Methods

Thirty-two healthy volunteers were recruited into the study. Each subject had two legs that were randomized into SCD or AFE. After 15 minutes of rest, SCD or AFE was applied, followed by 15 minutes of rest, and then an alternate treatment was given to the second leg. The sequence of leg and methodology was then reversed so the second treatment was applied to the first leg, and the first treatment to the second leg, and measurements were obtained. All treatments were separated by a rest period of 15 minutes. The venous velocity on each leg was measured by Doppler ultrasound at the superficial femoral vein. Venous velocity was measured initially (first cycle peak venous velocity) and during subsequent cycles. The alternate treatments on both legs with both treatments allowed for analysis in a manner where each subject could act as its control.

Results

Relative to baseline bed rest, SCD increased peak venous flow velocity by 112%, while AFE increased peak venous flow velocity by 161%. AFE resulted in 43% higher venous velocity on average than did SCDs (p<0.05).

Conclusions

AFE leads to significantly higher venous flow in the femoral veins of healthy subjects.

## Introduction

Deep vein thrombosis and pulmonary embolism are collectively referred to as venous thromboembolic events. Each year, four million surgical patients and eight million medical patients are hospitalized in the United States at moderate or high risk for venous thromboembolism (VTE) [1]. The four risk factors most predictive of VTE are previous VTE, bed rest, central venous catheter, and cancer [2]. A modeling study using the US Healthcare Cost and Utilization Project estimated about 200,000 VTE-related events among eight million acutely ill hospitalized medical service patients annually (2.5%) [3]. The frequency of VTE in hospitalized patients with cancer has been anywhere between 0.6% and 7.8% of symptomatic patients screened with ultrasound [4]. Two different techniques are employed to reduce VTEs prophylactically: mechanical and pharmacologic. Mechanical techniques work by reducing stasis and possibly by increasing systemic fibrinolysis [5-6]. Unfractionated heparin, when administered preoperatively and continued postoperatively, has been shown to be effective in preventing VTEs [7]; however, a major concern is intraoperative and postoperative bleeding, specifically hematoma formation and the need for monitoring for heparin-induced thrombocytopenia [8-9]. Low molecular weight heparin has more antifactor Xa and less antithrombin than unfractionated heparin and, while decreasing wound hematomas, has the same efficacy as unfractionated heparin [10]. Some data suggest that pneumatic compression is as effective as heparin in reducing deep vein thrombosis (DVT) incidence [11-13]. Another systematic review suggested that low molecular weight heparin may be superior to unfractionated heparin, with no significant difference between unfractionated heparin versus mechanical compressions [14]. Between 1979 and 1999, despite all the advances, the incidence of DVT in hospitalized patients increased from 0.8% to 1.3% of admissions [15]. The American College of Chest Physicians (ACCP) does not recommend pharmacologic prophylaxis over mechanical prophylaxis unless a documented hypercoagulable parameter is present. The American Society for Clinical Oncology recommends using pharmacologic methods of chemoprophylaxis over mechanical methods only in patients undergoing cancer surgery of > 30-minute duration unless a contraindication to pharmacologic methods is present [4]. The 2012 ACCP guidelines recommend mechanical methods of VTE prophylaxis in patients at high risk for bleeding complications until the risk of bleeding diminishes and early ambulation or pharmacological methods can be started [16].

Ambulation reduces VTE risk by decreasing stasis and the release of antithrombotic factors from the endothelial surface. Active ankle movements activate the calf muscles complex, thus promoting vein circulation and decreasing the risk of DVT [17]. During surgery or hospitalization, bed rest induces stasis. Mechanical compression devices compress the deep veins from the outside but do not elicit contraction of the muscles as does ambulation [18]. In addition, the compression obtained from a mechanical device induces a gentle increase in blood velocity in contrast to calf muscle contraction, which can cause turbulence. This turbulence induces significantly higher shear forces at the periphery of vessel walls. This physiologic difference in the mechanism of sequential compression versus ambulation led us to investigate an easy and cost-effective method to prevent VTE risk in hospitalized patients. We intended to measure venous velocity in the superficial femoral vein while using sequential compression devices as compared to flexion/extension while in bed.

## Materials and methods

We recruited 40 healthy volunteers for our study. The University of Tennessee’s Institutional Review Board (11-01465-XP) approved the study. Written informed consent was obtained. Healthy volunteers who were included were screened with a medical questionnaire, and anyone with a history of VTEs or any cardiac, vascular, diabetic, skin, neuropathic, morbid obesity (BMI>40), pregnancy, or orthopedic condition was excluded. Volunteers were fully ambulatory with a gynecologic oncology group performance score of zero. We had an attrition rate of eight volunteers due to scheduling conflicts. Demographic data, including age, sex, BMI, race, medical conditions, and previous surgeries, were collected. Unique identifiers were then destroyed, and data were collected in a secure spreadsheet.

A low-active lifestyle typically involves on average 6,000 steps a day [19]. Therefore, we sought to simulate walking while the volunteer was horizontal in bed. Such a low active ambulation state is considered protective for VTE risk [20]. Ankle flexion/extensions (AFE), were performed in bed at the rate of 30 flexions/extensions per minute (equivalent to walking at a speed of 1 mile in 20 minutes) [19]. The pneumatic compression device used was the A-V impulse system (Kendall Company, Mansfield, MA).

Peak velocity was measured in the upper superficial femoral veins bilaterally, just caudal to the common femoral veins, using a 6 MHz linear transducer (6L3 transducer) on an Acuson Sequoia 512 ultrasound machine (Siemens Medical Solutions USA, Inc., Mountain View, CA) with duplex color and spectral Doppler interrogation. The transducer frequency was set at 6 MHz for grayscale imaging and at 3.5 MHz for color and spectral Doppler imaging. The angle between the axis of flow and the Doppler beam was maintained at ≤60°. Transducer contact with the leg was kept as light as possible to exclude compression of the vein, which could spuriously elevate velocities. Peak velocity was measured electronically on the spectral Doppler display on the ultrasound machine at the time of the examination and recorded in a spreadsheet. A computer-generated program randomized volunteers’ right and left legs into an SCD arm or AFE arm. Moreover, a second randomization was made regarding where the first treatment was to be applied (e.g., SCD or AFE).

For every subject, the trial was started after resting for 15 minutes in the dorsal supine position, followed by measurement of baseline femoral vein velocity bilaterally. SCD or AFE was applied according to the randomization schema to the right or left leg, followed by a rest period of 15 minutes and then the alternate treatment (second treatment on the second leg). The sequence of leg and methodology was reversed, so the second treatment was applied to the first leg, and the first treatment to the second leg, and measurements were obtained. All treatments were separated by a rest period of 15 minutes to minimize any interference effect of one treatment into the other. The collected randomized data on both legs with both treatments allowed for analysis in a manner where each subject could act as its control (repeated measures analysis of variance).

In the SCD arm, the sequential compression device was placed on the leg. Initial pump compression was obtained. Then, after several pump cycle compressions according to the manufacturer’s specification, wave tracing of venous blood flow, consistent with pump activation, was recorded and stored in the ultrasound hard drive. Using the proprietary software of the ultrasound unit, the baseline venous velocity and peak velocity were measured three times, and mean values for baseline and peak femoral venous velocities were averaged. These measurements were obtained by an experienced radiology physician with more than 30 years of experience with ultrasound. Every effort was made to ensure that the probe was positioned consistently above the superficial femoral vein. The Doppler sample gate size matched the diameter of the vein. SCD devices were applied following the manufacturer’s guidelines.

Repeated measures univariate analysis of variance was then performed in a non-parametric fashion. For all test conditions, P=0.05. We chose to use repeated measures univariate analysis of variance as each subject and each extremity, even when randomized, had a pre- and post-velocity measurement for both SCD and AFE. In addition, having a baseline flow velocity provided a control. This approach made the statistics powerful by reducing error variance and providing controls. Baseline flow was recorded for all subjects before activating a device. In addition, the common femoral vein was checked for acute thrombosis, and none was found. Peak venous flow velocities were obtained after the first SCD compression and AFE and with continued SCD compressions and AFE (Table 1).

**Table 1:** All units in centimeters/second C.I. – confidence interval, Std. Dev. – Standard Deviation, Min. – Minimum, Max. – Maximum, AFE – Ankle flexion/extension, SCD – Sequential compression device, e – Initial activation of intervention

	Mean	Std. Dev.	C.I. of Mean	Min.	Max.	Median	25%	75%
Baseline	35.1	14.2	5.1	13.0	64.0	36.0	24.5	45.0
AFEe	123.0	37.6	13.5	35.2	205.0	129.0	96.0	144.5
AFE	91.8	40.9	8.6	22.3	124.0	82.1	66.5	95.2
SCDe	70.4	31.5	11.3	10.0	134.0	71.3	46.0	90.5
SCD	74.6	25.6	9.2	23.0	118.2	76.5	64.0	94.3

## Results

Augmentation of peak venous flow was seen with every intervention. Non-parametric methods were used to analyze the data. Friedman repeated measures analysis of variance on ranks was initially used to test differences between baseline, AFE, and SCD data. Average baseline venous flow velocity was measured at 35.1±14.2 cm/s (mean±SD) in bed rest volunteers. We found statistically significant differences in median values among the treatment groups (p<0.001, chi-square=102.4, df=4). Peak venous flow velocity after the first cycle of SCD was measured at 70.4±31.5 cm/s and after continued compressions at 74.6±25.6 cm/s. Peak venous flow velocity after the first cycle of AFE was measured at 123.0±37.6 cm/s and after continued cycles at 91.8±40.9 cm/s. Relative to baseline bed rest, SCD uses increased peak venous flow velocity by 112%, while AFE increased peak venous flow velocity by 161%. AFE resulted in 43% higher venous velocity on average than did SCDs (p<0.05).

To isolate the differences, we ran a post-hoc non-parametric repeated measures analysis of variance (Student-Newman-Keuls method) (Table 2). All treatment groups were significantly different from each other.

**Table 2:** All pairwise multiple comparison procedures on ranks (Student‐Newman‐Keuls method) Legends: AFE – Ankle flexion/extension, SCD – Sequential Compression Device, e – Initial activation of intervention, Diﬀ of Ranks – Diﬀerence of ranks, q – test statistic, p – the probability of obtaining a test statistic

	q	Diﬀ of Ranks	P<0.05
AFEe vs. Baseline	14.0	125.5	Yes
SCDe vs. Baseline	12.5	50	Yes
AFEe vs. SCDe	10.3	75.5	Yes
AFE vs. Baseline	10.2	74.5	Yes
SCD vs. Baseline	11.0	62.5	Yes
AFE vs. SCD	3.0	12.0	Yes
AFEe vs. SCD	11.1	63.0	Yes
AFE vs. SCDe	4.3	24.5	Yes

## Discussion

Many conditions can predispose people to stasis. Other factors that lead to thrombosis include endothelial injury and hypercoagulability. Following ACCP guidelines, we have used SCDs in our benign and oncology population to successfully reduce the risk of VTEs at a rate comparable to that seen in the ENOXACAN II study [21] in patients at low risk for VTEs or in patients at high risk for intra- and postoperative bleeding. We wanted to investigate femoral venous flow velocities after ankle flexion-extension instructions and compare them to velocities resulting from sequential compression devices. SCD use increased peak venous flow velocity by 112%, while AFE increased peak venous flow velocity by 161%. Both of these increases were statistically significant. In addition, AFE resulted in flow velocities 43% higher than in the SCD arm, which is also a significantly higher increase.

Cancer, stasis, and surgery are the main risk factors contributing to thromboembolic disease. Ambulation has been known to be effective in reducing the incidence of DVT. However, many bedridden patients may not achieve active ambulation, especially in the first day or two after surgery. Therefore, SCD is used in these bedridden patients to increase venous flow in the lower extremities to decrease the risk of DVT. In our study, we found that simple flexion/extension of the ankle produced an even higher femoral venous flow rate.

Li et al. performed a study on 193 patients undergoing orthopedic surgery and randomly assigned the individuals into two groups: the control group (N=97) received routine nursing care, while the case group (N=96) received routine nursing and active ankle movements (dorsiflexion, plantar flexion, and valgus flexion in sequence with a frequency of 30 times/minute at cycles of eight minutes (cycle every 30 minutes and 20 cycles a day). They measured the maximum venous outflow (MVO) and maximum venous capacity (MVC) via plethysmography. MVC and MVO on day 14 were significantly higher in the case group, and the case group had a significantly lower rate of DVT incidence than the control group. Their study result also shows the importance of ambulation in the prevention of DVT. Another positive aspect of their study was a longer follow-up to see and compare the incidence of DVT. However, the study was done on patients undergoing orthopedic surgery, and the study lacks a comparison of ankle flexion movements with SCDs [17].

Our study has several limitations. First, we used normal volunteers instead of patients with risk factors contributing to DVT. Indeed, this is our preliminary study evaluating femoral venous flow, not DVT. In future studies, we will conduct a randomized trial of AFE risk versus SCD versus heparin in reducing DVT rate. Second, our study did not establish a reduced DVT rate. Indeed, the study was designed to evaluate femoral venous flow, which is a surrogate marker for improved vascular flow. The study supported the hypothesis that AFE improves venous vascular flow. The third potential criticism of the study is that AFE is difficult to implement due to variable patient compliance. Indeed, SCD has also been observed not to be worn by patients on bed rest. Our concurrent study comparing a period before the implementation of AFE showed a rate of DVT in postoperative patients with uterine cancer of 4.2% [21]. Subsequent studies on the same population but implementing AFE instructions to these patients resulted in a 0.4% DVT rate in postoperative patients with uterine cancer [22].

## Conclusions

AFE seems to be more effective than SCD in increasing femoral venous flow. Most studies done on the effect of active ankle movements are on the patient population undergoing orthopedic surgery, and most of them are more focused on the effect of active ankle movement rather than comparing AFE vs SCD alone. Future studies should randomize AFE versus SCD in reducing the DVT rate in postoperative gynecologic oncology patients.
